# Plasma mammalian leptin analogue predicts reproductive phenology, but not reproductive output in a capital‐income breeding seaduck

**DOI:** 10.1002/ece3.4873

**Published:** 2019-01-13

**Authors:** Holly L. Hennin, Pierre Legagneux, H. Grant Gilchrist, Joël Bêty, John P. McMurtry, Oliver P. Love

**Affiliations:** ^1^ Department of Biological Sciences University of Windsor Windsor Ontario Canada; ^2^ CNRS – Centre d'Etudes Biologique de Chizé Villiers‐en‐bois France; ^3^ Département de biologie et Centre d'etudes nordiques Université Laval Québec City Quebec Canada; ^4^ Environment and Climate Change Canada National Wildlife Research Centre, Carleton University Ottawa Ontario Canada; ^5^ Départment de Biologie, chimie et géographie and Centre d’études nordiques Université du Québec à Rimouski Rimouski Quebec Canada; ^6^ Southern Plains Agricultural Research Center United States Department of Agriculture College Station Texas

**Keywords:** clutch size, common eider, energetic demand, leptin, plasma mammalian leptin analogue, reproductive phenology, reproductive success

## Abstract

To invest in energetically demanding life history stages, individuals require a substantial amount of resources. Physiological traits, particularly those related to energetics, can be useful for examining variation in life history decisions and trade‐offs because they result from individual responses to environmental variation. Leptin is a protein hormone found in mammals that is proportional to the amount of endogenous fat stores within an individual. Recently, researchers have confirmed that a mammalian leptin analogue (MLA), based on the mammalian sequence of leptin, is present with associated receptors and proteins in avian species, with an inhibitory effect on foraging and body mass gain at high circulating levels. While MLA has been both quantified and manipulated in avian species, little is currently known regarding whether plasma MLA in wild‐living species and individuals is associated with key reproductive decisions. We quantified plasma MLA in wild, Arctic‐nesting female common eiders (*Somateria mollissima*) at arrival on the breeding grounds and followed them to determine subsequent breeding propensity, and reproductive phenology, investment, and success. Common eiders are capital‐income breeding birds that require the accumulation of substantial fat stores to initiate laying and successfully complete incubation. We found that females with lower plasma MLA initiated breeding earlier and in a shorter period of time. However, we found no links between plasma MLA levels and breeding propensity, clutch size, or reproductive success. Although little is still known about plasma MLA, based on these results and its role in influencing foraging behaviors and condition gain, plasma MLA appears to be closely linked to reproductive timing and is therefore likely to underlie trade‐offs surrounding life history decisions.

## 1 INTRODUCTION

Life history trade‐offs can be generated through the constraints of allocating limited resources to multiple competing life history traits and decisions (McNamara & Houston, 1996; Stearns, 1989). As such, individuals that are capable of obtaining more resources and optimizing resource allocation across all energetic demands are predicted to relax the associated trade‐offs and maximize success (Kisdi, Meszéna, & Pásztor, 1998; McNamara & Houston, 1996; Rowe, Ludwig, & Schluter, 1994; Stearns, 1992). For example, individuals of migratory species that arrive on the breeding grounds in greater condition, or gain in condition more quickly once they arrive, can invest in reproduction earlier (Hennin et al., 2016) and invest in a greater number of offspring (Bêty, Gauthier, & Giroux, 2003; Descamps, Bêty, Love, & Gilchrist, 2011; Gladbach, Gladbach, & Quillfeldt, 2010; Lepage, Gauthier, & Menu, 2000). Physiological traits are excellent candidates for examining variation in life history traits since they can be mechanistic drivers of individual state and are therefore at the center point of many life history trade‐offs (Ketterson & Nolan, 1999; Ricklefs & Wikelski, 2002; Williams, 2008; Zera & Harshman, 2001). In particular, traits related to individual state or condition may be useful for understanding variation underlying investment in energetically demanding life history stages (Cornelius, Boswell, Jenni‐Eiermann, Breuner, & Ramenofsky, 2013; Hennin et al., 2015; Love, Madliger, Bourgeon, Semeniuk, & Williams, 2014).

Leptin is a mammalian protein hormone produced by adipocytes which signals to the hypothalamus the level of fat stores an individual has, with downstream roles in appetite control (Frederich et al., 1995; Friedman & Halaas, 1998), regulating fattening (Kochan, Karbowska, & Meissner, 2006; Schradin, Raynaud, Arrivé, & Blanc, 2014), and influencing maternal investment in reproduction (French, Greives, Zysling, Chester, & Demas, 2009). Currently, there is a growing body of evidence that a leptin‐like analogue exists in avian species despite controversy over its existence and terminology (Prokop, Duff, Ball, Copeland, & Londraville, 2012; Seroussi et al., 2016; Sharp, Dunn, & Waddington, 2008; Simon, Rideau, & Taouis, 2009). For instance, leptin receptors based on mammalian sequences have been identified in multiple tissue types in chicken (*Gallus gallus*; Taoius et al., 2001, Paczoska‐Eliasiewicz et al., 2006, Ohkubo, Nishio, Tsurudome, Ito, & Ito, 2007), leptin protein sequences have been identified in a number of avian species (Prokop et al., 2014), and leptin gene expression has been identified in avian adipose and liver tissues (Quillfeldt, Everaert, Buyse, Masello, & Dridi, 2009; Taouis et al., 1998). Given that this trait and its associated receptors have been identified based on mammalian leptin sequences, it has been suggested that these studies have instead quantified a mammalian leptin analogue (MLA) rather than true avian leptin (Seroussi et al., 2016).

Given the diverse tissues in which plasma MLA and associated receptors have been identified, and that previous research indicates that plasma MLA in avian species is related to energetics and energetic management it is likely that this trait plays a role in influence reproductive traits. For example, manipulations administering mammalian leptin to avian species have been shown to *decrease* foraging behavior in blue tits (*Cyanistes caeruleus*, Lõhmus, Sundström, Halawani, & Silverin, 2003) and has resulted in *decreased* body mass in Asian blue quail (*Coturnix chinensis*, Lõhmus, Sundström, & Silverin, 2006). Circulating plasma MLA in European starlings (*Sturnus vulgaris*) has been shown to change with reproductive stage (higher plasma MLA in egg‐laying and clutch completion, and lower plasma MLA during incubation and chick rearing), likely in response to changing energetic demands across the reproductive period (Kordonowy, McMurtry, & Williams, 2010). Finally, experimental elevations of mammalian leptin in wild‐living great tits (*Parus major*) resulted in a higher probability of investing in a second brood and has therefore been hypothesized to interact with the hypothalamic–pituitary–gonadal (HPG) axis (Lõhmus & Björklund, 2009; but see te Marvelde & Visser, 2012). As such, elevations of plasma MLA may indicate availability of high endogenous adipose stores and appears to play a biologically relevant role in not only affecting foraging and resource acquisition, but also in indirectly impacting fitness‐related traits.

Here, we quantify plasma levels of this energetic trait with structural homology to mammalian leptin (plasma MLA) in pre‐laying Arctic‐nesting, female common eiders (*Somateria mollissima*), to determine whether individual variation in plasma MLA can predict inter‐individual variation in the decision to invest in reproduction (breeding propensity), reproductive phenology (relative laying date and delay before laying), reproductive investment (clutch size), and reproductive success (hatching success). Arctic‐nesting common eiders have a mixed (capital‐income) reproductive strategy, using a combination of stored, endogenous fat as well as incoming resources from foraging on the breeding grounds to fuel their follicle growth (Sénéchal, Bêty, Gilchrist, Hobson, & Jamieson, 2011; Stephens, Boyd, McNamara, & Houston, 2009). Pre‐laying females must accumulate a significant amount of fat stores to initiate reproduction (Hennin et al., 2015; Sénéchal et al., 2011), fuel follicle growth, and ensure they still have enough remaining fat stores to successfully complete their 24‐day incubation fast (Bottitta, Nol, & Gilchrist, 2003; Sénéchal et al., 2011). Therefore, the accumulation and careful management of fat stores are critical to optimizing reproductive decisions, and maximizing fitness in this species (Descamps et al., 2011; Hennin et al., 2015; Jean‐Gagnon et al., 2018). Given that experimental elevations of plasma MLA have been shown to reduce foraging behavior and body mass in avian species (Cerasale, Zajac, & Guglielmo, 2011; Lõhmus et al., 2003, 2006), female eiders with higher plasma MLA may also exhibit reduced foraging behavior, gaining in fat stores, and body mass at a slower rate compared to females with lower plasma MLA. We therefore predict that pre‐laying females with higher plasma MLA will have slower rates of gain in fat stores, thereby exhibiting reduced breeding propensity (probability of reproducing), delayed breeding phenology (later lay date, longer delays prior to laying), and reduced investment in reproduction (smaller clutch sizes and lower reproductive success).

## 2 MATERIALS AND METHODS

### 2.1 Field methods

Our study colony is located at Mitivik Island (64°02′N, 81°47′W), a small, low‐lying island in a shallow productive bay in Nunavut, Canada. In 2006 and 2007, common eider females were captured opportunistically (*n* = 377) using flight nets from mid‐June to early July. This period of time coincides with the timing of arrival on the breeding grounds and the pre‐laying period (Descamps et al., 2011; Hennin et al., 2015; Jean‐Gagnon et al., 2018), making the timing of capture our best estimate of individual arrival date at the breeding grounds (Descamps et al., 2011). Within 3 min of capture, females were blood sampled from the tarsal vein with a 1 ml heparinized syringe and 23 G thin wall, 0.5‐inch needle. Samples were transferred to a heparinized eppendorf tube, kept cool and centrifuged at 10,000 rpm for 10 min within 4 hr of collection. The plasma was siphoned off and stored separately from the red blood cells at −80°C until further analysis.

After blood sampling, females were banded, weighed (g), and given a unique combination of shaped and colored nasal tags attached using UV degradable filament, followed by release. These nasal tags fall off at the end of the season, but allow us to easily track individuals through their reproductive period using spotting scopes from one of seven permanent blinds located around the periphery of the island. Twice daily, trained observers would scan the colony for nasal‐tagged females from seven permanent blinds with one blind dedicated solely to the searching and tracking of nasal‐tagged females specifically, and record nesting behaviors to accurately determine laying date and assess breeding stages of females. Once a female began laying, she was monitored twice daily to track incubation and reproductive success. We confirmed clutch sizes for females by crawling into the colony in transects across it to check individual nests. Since these checks generate significant disturbance, often resulting in the predation of focal common eider nests by nesting pairs of herring gulls (*Larus argentatus*) on the island, we recorded clutch sizes of focal females opportunistically. Therefore, we obtained clutch size for many birds (Table 1), but our analyses for clutch size nonetheless include fewer females than other reproductive metrics.

**Table 1:** Statistical summary of variables included in analyses, split by year and breeding stage, of common eider females nesting at Mitivik Island

Breeding stage	Year	Ordinal capture date			Body mass (g)			Plasma MLA (ng/ml)			Ordinal lay date			Clutch size		
		*N*	Mean	*SE*	*N*	Mean	*SE*	*N*	Mean	*SE*	*N*	Mean	*SE*	*N*	Mean	*SE*
Non‐breeder	2006	49	168.4	0.5	49	2,153.5	32.5	49	8.08	0.42	n/a	n/a	n/a	n/a	n/a	n/a
Non‐breeder	2007	71	171.7	0.2	71	1,985.2	25.9	71	9.69	0.32	n/a	n/a	n/a	n/a	n/a	n/a
Pre‐recruiting	2006	39	166.8	0.5	39	2,182.8	32.9	39	8.19	0.36	39	179.9	0.8	7	2.43	0.36
Pre‐recruiting	2007	66	172.2	0.2	66	2,175.5	22.4	66	9.69	0.34	66	185.0	0.4	15	3.13	0.24
RFG	2006	38	167.4	0.5	38	2,272.3	25.1	38	6.94	0.41	38	171.1	0.5	13	2.75	0.23
RFG	2007	25	172.9	0.3	25	2,215.6	22.8	25	8.02	0.54	25	177.4	0.5	5	4.00	0.55

### 2.2 Assay for mammalian leptin analogue

Assays for plasma mammalian leptin analogue were conducted in 2007 at the United States Department of Agriculture Laboratories (USDA‐Beltsville, MD, USA) by in‐house radioimmunoassay in tandem with samples from and following the methodologies outlined in Kordonowy et al. (2010). Briefly, putative recombinant chicken leptin (rcleptin) was provided by A. Gertler (Raver et al., 1998) and iodinated to a specific activity of 50 Ci/g, and then stored at −80°C until further use (Kordonowy et al., 2010). The primary antibody, rabbit anti‐rcleptin (Alpha Diagnostic International, San Antonio, TX) and second antibody, a sheep anti‐rabbit gamma globulin (Linco, Inc., St. Charles, MO) were both commercially available. Both primary and secondary antibodies were diluted using sodium phosphate buffer (0.05 M phosphosaline, pH 7.4) containing 0.025 M EDTA plus 0.05% Triton X‐100 (Sigma Chemical Co.) to a 1:1,600 and a 1:10 dilution, respectively. Plasma samples and radio‐labeled rcleptin (6000 c.p.m. I‐125‐labeled rcleptin; tracer) were diluted in 1% BSA phosphate buffer. These assays were run under nonequilibrium conditions.

On the first day of the assay, 100 μl of RIA diluent were added to a plastic tube, along with either 100 μl of standard or plasma, then vortexed and left overnight at 4°C. The following day, 100 μl of tracer was added to each tube and incubated at 4°C overnight once more. On the third day, 100 μl of the second antibody and carrier (normal rabbit serum, Linco, Inc., St. Charles, MO; 1:200 dilution in phosphate buffer) each were added to every tube, vortexed, and incubated at 4°C overnight a final time. On the final day of the assay, all tubes but the total count tubes were centrifuged at 2,500 rpm, the supernatant was aspirated and the remaining pellet was counted in a gamma counter. The RIA data reductions used log/logit transformations. The displacement curve using this assay method in wild birds has been tested previously and shown to be parallel to the standard curve with a sensitivity of 300 pg/tube and 96.1% recovery rate (Kordonowy et al., 2010). Due to the plasma volume required for this assay (~125 μl), we were only able to assay single samples and therefore could not calculate the amount of variation within a plate. However, the same assay conducted at this same institution has previously demonstrated low intra‐assay (3.2%) and inter‐assay coefficients of variation (5.1%; Kordonowy et al., 2010).

### 2.3 Identification of plasma mammalian leptin analogue

The gene sequence used to raise antibodies to quantify MLA in the current study was based on a mammalian sequence (Raver et al., 1998; Taouis et al., 1998), and therefore not representative of the actual avian leptin sequence (Seroussi et al., 2016), but rather a mammalian leptin analogue in an avian species. To identify what our assay may have been measuring, we ran a BLAST (basic local alignment search tool; Altschul, Gish, Miller, Myers, & Lipman, 1990) of the known mammalian leptin gene sequence open to all known avian genome sequences. Specifically, we ran both a BLASTx and BLASTn to identify possibly proteins and nucleotide sequences, respectively. We also ran a BLASTx and BLASTn of the known mammalian‐based primers used to raise our antibodies for our assays against all known avian genome sequences. In both instances, we found no supported matches within the current sequenced avian genome and cannot currently report an official name and function for this quantified trait.

### 2.4 Statistical analyses

Since different breeding stages have differing energetic demands (Hennin et al., 2015; Sénéchal et al., 2011), which may influence the relationship between plasma MLA and reproductive parameters, we first split individuals into three categories for analyses (Hennin et al., 2016, 2015). Females that were captured, but never detected as a breeder (i.e., not resighted on a nest) that year at the colony were considered non‐breeders (Jean‐Gagnon et al., 2018). Given that common eiders are philopatric and colonial nesters, and that this colony is the only one that is within 200 km (Jean‐Gagnon et al., 2018), it is unlikely that females were misclassified as non‐breeders and instead nested elsewhere. Breeding individuals were subsequently split into two breeding stages based on the number of days between capture and laying. Females were categorized as *pre‐recruiting* (i.e., not yet growing follicles for laying) if they were 8 days or longer away from laying when captured, or were categorized as *rapid follicle growth* (*RFG*, i.e., quickly growing follicles in preparation for laying, Williams, Kitaysky, & Vézina, 2004) if they were 1–7 days away from laying at capture (Hennin et al., 2015). These categories are based on the average duration of follicle growth in common eiders (approx. 6 days, Alisauskas & Ankney, 1992) plus an additional 28 hr required to lay an egg (Watson, Robertson, & Cooke, 1993). There were no instances of recapturing individuals and therefore there are no repeated samples from individuals.

Previous research at this colony has shown that correcting for body size only enhances our ability to explain variation in body condition by roughly 3%, therefore body mass on its own is an accurate measure of body condition (Descamps et al., 2011). Rather than analyze ordinal dates for arrival and laying, we use relative dates (individual arrival or lay date relative to the median of the colony for each year; Lepage et al., 2000) to help control for additional annual variation that may be attributable to environmental variation (i.e., two extreme years in terms of weather, ice break‐up, resource availability) and better focus the analysis on individual‐based differences. To test for differences in body mass and plasma MLA across breeding stages and years, we ran a two‐way ANOVA for each variable including year, breeding stage, and a year by breeding stage interaction, followed by Tukey post hoc tests with a Bonferroni correction. We then tested for correlations between body mass and plasma MLA within each of the three breeding stages with a Bonferroni correction. We analyzed our reproductive parameters using either general linear models (relative lay date, delay before laying) or generalized linear models (clutch size, reproductive success) depending on our dependent variables. In the analyses for the delay before laying and relative laying date, plasma MLA, year, body mass, and relative capture date were included as fixed effects. For clutch size and reproductive success analyses, we included the same fixed effects, but exchanged relative capture date for relative laying date due to the known influence of laying date on both clutch size and reproductive success (Descamps et al., 2011). We tested for the interaction between body mass and plasma MLA given that plasma MLA is related to foraging and fattening (Lõhmus et al., 2003, 2006); however, in all analyses this interaction was nonsignificant and it was therefore removed. Breeding propensity analyses included only non‐breeding and pre‐recruiting females because females in RFG were already committed to laying. All samples were independent of each other. Although we attempted to collect as many variables from each female as possible, we were unable to obtain every variable from every female, therefore making our sample sizes across analyses variable. As such, each reproductive analysis required a different subset of individuals, making each data set unique and unaffected by issues of multiple testing. All results are reported as means ± *SEM* unless otherwise stated. Our analyses were run in JMP 12.0.1.

## 3 RESULTS

**Figure 1:**
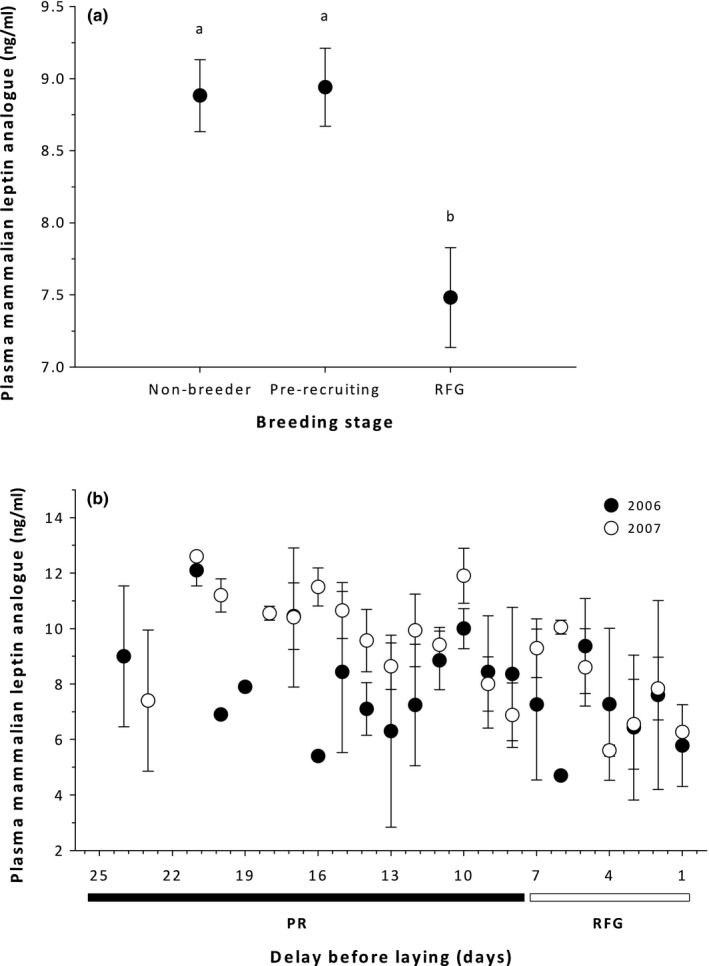
Differences in plasma mammalian leptin analogue (MLA) across breeding stages (a), and changes in plasma mammalian leptin analogue across days in the pre‐recruiting and rapid follicle growth (RFG) periods (b) in Arctic‐nesting, female common eiders. Different letters indicate significant differences between groups

Year (*p* = 0.002), breeding stage (*p* < 0.0001), and their interaction (*p* = 0.01) significantly predicted body mass (*F* = 8.10_5,282_, *p* < 0.0001; Table 1) with non breeders in 2007 having lower body masses compared to all other stages in all other years. There were significant differences in plasma MLA between years (*p* < 0.0001; 2006: 7.73 ± 0.24 ng/ml, *n* = 126; 2007: 9.13 ± 0.24 ng/ml, *n* = 162) and breeding stages (*p* = 0.002), but not their interaction (*p* = 0.82), with RFG females having lower plasma MLA than pre‐recruiting or non‐breeding females (*F* = 8.10_5,282_, *p* < 0.0001; Figure 1). We found that body mass and plasma MLA were negatively correlated in non‐breeding birds (*r* = −0.26, *p* = 0.004, *n* = 120), but uncorrelated in pre‐recruiting (*r* = −0.01, *p* = 0.92, *n* = 105) or RFG females (*r* = −0.05, *p* = 0.66, *n* = 63). Plasma MLA did not predict breeding propensity, after controlling for the known effect of body mass (Table 2), with breeding birds having higher pre‐laying body masses (2,178.2 ± 18.5 g) than non‐breeders (2,053.9 ± 21.6 g).

**Table 2:** Summary of fixed effects and parameter estimates from breeding propensity analyses of common eider females

Variable	Fixed effects			Parameter estimates			
	*χ* ^2^	*df*	*p*	Estimate	*SE*	*χ* ^2^	*p*
Intercept	n/a	n/a	n/a	6.586	1.689	17.6	**<0.0001**
Year	2.35	1	0.13	0.236	0.155	2.35	0.13
Body mass	18.09	1	**<0.0001**	−0.003	0.001	18.08	**<0.001**
Relative capture date	0.82	1	0.36	0.050	0.056	0.823	0.36
Plasma MLA	0.45	1	0.51	−0.036	0.053	0.45	0.51

Note

Bolded *p*‐values indicate significant effects.

Plasma MLA significantly predicted variation in both the delay before laying and relative laying date for both pre‐recruiting and RFG females, after controlling statistically for the effect of body mass or relative capture date (Tables 3 and 4). Females with higher plasma MLA had longer delays before laying and later relative lay dates compared to females with lower plasma MLA. However, there was no predictive capacity for variation in plasma MLA on clutch size or reproductive success, regardless of breeding stage (Tables 5 and 6).

**Table 3:** Summary of fixed effects for delay before laying and relative lay date analyses in pre‐recruiting and rapid follicle growth (RFG) common eider females

Breeding stage	Analysis	Variable	*F*	*df*	*p*
Pre‐recruiting	Delay before laying	Year	0.30	1	0.58
		Body mass	13.00	1	**0.0005**
		Relative capture date	2.92	1	0.09
		Plasma MLA	3.97	1	**0.05**
	Relative laying date	Year	73.49	1	**<0.0001**
		Body mass	13.01	1	**0.0005**
		Relative capture date	29.58	1	**<0.0001**
		Plasma MLA	3.97	1	**0.05**
RFG	Delay before laying	Year	2.63	1	0.11
		Body mass	0.94	1	0.34
		Relative capture date	5.51	1	**0.02**
		Plasma MLA	4.64	1	**0.04**
	Relative laying date	Year	95.35	1	**<0.0001**
		Body mass	0.94	1	0.34
		Relative capture date	59.72	1	**<0.0001**
		Plasma MLA	4.64	1	**0.04**

Note

Bolded *p*‐values indicate significant effects.

**Table 4:** Parameter estimates of fixed effects presented in Table 3, for delay before laying and relative lay date analyses in pre‐recruiting and rapid follicle growth (RFG) common eider females

Breeding stage	Variable	Delay before laying				Relative laying date			
		Estimate	*SE*	*t*	*p*	Estimate	*SE*	*t*	*p*
Pre‐recruiting	Intercept	24.221	3.994	6.06	**<0.0001**	16.221	3.994	4.06	**<0.0001**
	Year	0.206	0.374	0.55	0.58	3.206	0.374	8.57	**<0.0001**
	Body mass	−0.006	0.002	−3.61	**0.0005**	−0.006	0.002	−3.61	**0.0005**
	Relative capture date	−0.239	0.140	−1.71	0.09	0.761	0.140	5.44	**<0.0001**
	Plasma MLA	0.263	0.132	1.99	**0.05**	0.263	0.132	1.99	**0.05**
RFG	Intercept	6.517	4.045	1.61	0.11	−1.480	4.047	−0.37	0.71
	Year	−0.427	0.263	−1.62	0.11	2.573	0.263	9.76	**<0.0001**
	Body mass	−0.002	0.002	−0.97	0.34	−0.002	0.002	−0.97	0.34
	Relative capture date	−0.233	0.099	−2.35	**0.02**	0.767	0.099	7.73	**<0.0001**
	Plasma MLA	0.201	0.093	2.15	**0.04**	0.201	0.093	2.15	**0.04**

Note

Bolded *p*‐values indicate significant effects.

**Table 5:** Summary of effect tests for clutch size and reproductive success analyses in pre‐recruiting and rapid follicle growth (RFG) common eider females

Breeding stage	Analysis	Variable	*χ* ^2^	*df*	*p*
Pre‐recruiting	Clutch size	Year	0.28	1	0.59
		Body mass	0.68	1	0.41
		Relative lay date	0.49	1	0.48
		Plasma MLA	0.19	1	0.67
	Reproductive success	Year	11.43	1	**0.0007**
		Body mass	0.34	1	0.56
		Relative lay date	32.33	1	**<0.0001**
		Plasma MLA	0.44	1	0.51
RFG	Clutch size	Year	0.52	1	0.47
		Body mass	0.19	1	0.66
		Relative lay date	0.34	1	0.56
		Plasma MLA	0.42	1	0.52
	Reproductive success	Year	0.03	1	0.87
		Body mass	0.02	1	0.89
		Relative lay date	1.17	1	0.27
		Plasma MLA	0.16	1	0.69

Note

Bolded *p*‐values indicate significant effects.

**Table 6:** Parameter estimates of fixed effects presented in Table 3 for clutch size and reproductive success analyses in pre‐recruiting and rapid follicle growth (RFG) common eider females

Breeding stage	Variable	Clutch size				Reproductive success			
		Estimate	*SE*	*χ* ^2^	*p*	Estimate	*SE*	*χ* ^2^	*p*
Pre‐recruiting	Intercept	−0.535	2.225	0.06	0.81	−2.035	3.011	0.46	0.5
	Year	−0.088	0.167	0.28	0.59	−1.059	0.344	11.44	**0.007**
	Body mass	0.001	0.001	0.68	0.41	0.001	0.001	0.34	0.56
	Relative lay date	−0.032	0.046	0.49	0.49	0.397	0.088	32.33	**<0.0001**
	Plasma MLA	−0.025	0.059	0.18	0.67	−0.066	0.100	0.44	0.51
RFG	Intercept	−0.527	2.811	0.04	0.85	−1.437	4.581	0.10	0.75
	Year	−0.125	0.173	0.52	0.47	0.060	0.373	0.03	0.87
	Body mass	0.001	0.001	0.19	0.66	0.000	0.002	0.02	0.89
	Relative lay date	−0.028	0.049	0.34	0.56	0.114	0.106	1.18	0.28
	Plasma MLA	0.045	0.070	0.42	0.52	0.043	0.110	0.15	0.70

Note

Bolded *p*‐values indicate significant effects.

## 4 DISCUSSION

Currently, little is known about plasma mammalian leptin analogue (MLA) in avian species (Cerasale et al., 2011; Kordonowy et al., 2010; Quillfeldt et al., 2009), and to date, no studies have examined how variation in plasma MLA relates to reproductive decisions, investment, or reproductive success in wild birds. We demonstrate that plasma MLA differs among years and reproductive stages in arctic‐nesting common eiders, and present the first evidence that plasma MLA significantly predicts variation in reproductive phenology in pre‐laying females. Consistent with our predictions, we show that birds with higher pre‐laying MLA delayed the start of breeding (longer delays before laying and later lay dates). However, plasma MLA did not predict breeding propensity, clutch size, or reproductive success. This was surprising considering how important the timely acquisition of fat stores is to common eiders prior to reproducing, with impacts on reproductive investment and reproductive success (Bêty et al., 2003; Descamps et al., 2011; Hennin et al., 2016; Lepage et al., 2000).

### 4.1 Variation in plasma mammalian leptin analogue

We found individual‐based variation in female common eiders in plasma MLA across breeding stages (Figure 1a,b). To date only been a handful of studies that have examined natural variation in this trait, including in thin‐billed prion chicks (Quillfeldt et al., 2009, ~1.7–2.9 ng/ml), domestic Thai chickens (*Gallus domesticus*) across the reproductive period (Ngernsoungnern et al., 2012, ~0.05–1.15 ng/ml), and European starlings within and across life history stages (~10–32 ng/ml, Kordonowy et al., 2010). We found that common eiders have a range of plasma MLA values (2–15 ng/ml, this study) more similar to those of European starlings, likely because these are wild individuals not selected for growth rate, unlike domesticated species (Lõhmus et al., 2006; Ngernsoungnern et al., 2012) in which the selective forces driving foraging are more relaxed. Further, the eiders in this study were in adult life history stages likely under different energetic requirements than thin‐billed prion chicks, highlighting the context‐dependent nature of interpreting variation in plasma MLA within and across species.

Although plasma MLA in *domestic, agricultural* avian species has been linked to endogenous fat stores and body mass (Lõhmus & Sundström, 2004; Lõhmus et al., 2006, we found that in pre‐recruiting and RFG eider females, body mass, and plasma MLA were not correlated. This is consistent with findings in other *wild *avian species (Kordonowy et al., 2010; Quillfeldt et al., 2009) which often report that plasma MLA levels are often disassociated with body mass in life history stages that require fattening (e.g., migration: Townsend, Kunz, & Widmaier, 2008; Gogga, Karbowska, Kochan, & Meissner, 2013). Considering that pre‐laying common eiders are in a life history stage in which they must obtain a substantial amount of endogenous fat resources quickly, plasma MLA levels may therefore be disassociated with body mass, thus explaining the lack of both correlation and interactive effects in all of our analyses. Interestingly, despite the lack of association between plasma MLA and body mass, plasma MLA could significantly predict reproductive decisions of pre‐recruiting and RFG females.

Finally, plasma MLA was higher in non‐breeding and pre‐recruiting females compared to RFG females. Previous research in common eiders has shown that physiological fattening rates (measured though triglycerides) follow similar patterns with females in RFG having lower physiological fattening (Hennin et al., 2015). Overall, females eiders thus have lower plasma MLA (this study) and physiological fattening during the RFG period compared to the pre‐recruiting period despite the energetic demands of growing follicles (Hennin et al., 2015). Since female eiders need to achieve a minimum body mass to initiate follicle growth (Descamps et al., 2011; Sénéchal et al., 2011), and all RFG females should have achieved this threshold body mass, the reduction in plasma MLA during the RFG period may represent the change in females from rapid and timely somatic fattening, toward more minimal foraging to top up resources available for follicle growth without impacting body condition. Pre‐recruiting females captured shortly after their arrival on the breeding grounds may thus have high plasma MLA because they are depositing significant fat stores to recover from migration and prepare for reproduction.

### 4.2 Influence of plasma mammalian leptin analogue on reproductive parameters

In both pre‐recruiting and RFG females, individuals with higher plasma MLA had longer delays before laying once on the breeding grounds (i.e., longer time interval between arrival and laying date) and had later laying dates. This occurred despite the lack of association between body mass and plasma MLA, potentially indicating that there may be other indirect impacts of plasma MLA on energetics and energetic management besides direct impacts on body mass. In avian species, experimental manipulations of plasma MLA, using mammalian leptin, have been shown to reduce foraging behaviors (Lõhmus & Sundström, 2004; Lõhmus et al., 2003, 2006). Further, in wild‐breeding great tits (*Parus major*) females administered mammalian leptin had a higher likelihood of investing in a second brood (Lõhmus and Bjorklund, 2009, *but see* te Marvelde & Visser, 2012). If plasma MLA provides a signal of condition or endogenous fat stores and negatively influences foraging (Lõhmus & Sundström, 2004; Lõhmus et al., 2003, 2006), female eiders with high plasma MLA may have reduced foraging behavior, take longer to gain in condition for reproduction, and therefore lay later in the breeding season. Considering that plasma MLA influences the functioning of cultured ovarian cells (Sirotkin & Grossmann, 2007), advances the onset of puberty in chicken (Paczoska‐Eliasiewicz et al., 2006), and affects reproduction (Lõhmus and Bjorklund, 2009, this study), plasma MLA may be an important trait mediating life history decisions and potentially trade‐offs.

However, if high concentrations of plasma MLA indicate high endogenous fat stores then it seems counterintuitive that female eiders with higher concentrations of plasma MLA delayed laying for a longer period of time and laying later in the breeding season, rather than earlier. Although mammalian leptin has an endocrine function, true avian leptin has been found to fit more so within autocrine/paracrine function (Seroussi et al., 2016). It may be that plasma MLA may have a similar autocrine/paracrine function as in avian leptin, thus explaining its disassociation with body mass, and the inverse relationships between plasma MLA concentrations and reproductive phenology. Regardless, as in other physiological traits (Williams, 2008), there is still a substantial amount of diversity in the inherent ability of individuals to produce plasma MLA (Quillfeldt et al., 2009; Kordonowy et al., 2010, this study), and likely variation in individual sensitivity to the trait. Furthermore, although receptors for this trait have been identified in multiple avian tissues (Ohkubo et al., 2007; Paczoska‐Eliasiewicz et al., 2006; Taoius et al., 2001), the variation in receptor densities across tissues seasonally has yet to be documented. Research in other hormones suggests seasonal variation in receptor density is probable (e.g., androgens and estrogens: Fusani, Van't Hof, Hutchinson, & Gahr, 2000, corticosterone: Breuner & Orchinik, 2001), and therefore a likely contributing factor to individual and species variation in plasma MLA. Since plasma MLA appears to be related to energetics and energetic management, it is possible that some of this variability is also generated from environmental factors such as food availability (Schradin et al., 2014) or types of food consumed (Frederich et al., 1995), as is the case with leptin in mammalian species.

## 5 CONCLUSION

Recent evidence suggests that previous attempts to identify and quantify leptin in avian species have failed to identify true avian leptin because previous attempts have been based on mammalian leptin sequences (Seroussi et al., 2016). Despite these studies not quantifying true avian leptin, there is substantial evidence that avian species have receptors (Ohkubo et al., 2007; Paczoska‐Eliasiewicz et al., 2006; Taoius et al., 2001), plasma variation (Kordonowy et al., 2010; Ngernsoungnern et al., 2012; Quillfeldt et al., 2009, this study), proteins (Prokop et al., 2014), and gene expression (Quillfeldt et al., 2009; Taouis et al., 1998) related to mammalian leptin. Furthermore, individuals respond behaviorally, in body condition, and alter reproductive parameters in response to exogenous administration of mammalian leptin. Indeed, we found that variation in pre‐breeding plasma MLA related to timing of reproduction, implying that it may be an important mechanism underlying life history decisions. We therefore argue that plasma MLA appears to be an important trait mediating reproductive investment, and advocate that future research aims to identify this potentially important physiological trait.

## CONFLICT OF INTEREST

None declared.

## AUTHOR CONTRIBUTIONS

The original ideas were developed by OPL, JB, and HGG. The data were collected by OPL, and samples were assayed by JPM. The data were analyzed by HLH with input from OPL, PL, and JB. The manuscript was written by HLH with input from all co‐authors.

## Data Availability

Data has been archived in the Dryad repository: https://doi.org/10.5061/dryad.283n1c6 (Hennin et al. 2018).
